# The shifts of submucosal microbiota in treated patients with aggressive periodontitis

**DOI:** 10.3389/froh.2026.1855289

**Published:** 2026-09-22

**Authors:** Qiao Shi, Wenmin Zeng, Yu Cai, Qingxian Luan, Jing Zang, Xiaoqian Yu, Guojing Liu

**Affiliations:** 1 Beijing Key Laboratory of Digital Stomatology, NHC Key Laboratory of Digital Stomatology & NMPA Key Laboratory for Dental Materials, Department of Periodontology, National Center for Stomatology, National Clinical Research Center for Oral Diseases, National Engineering Research Center of Oral Biomaterials and Digital Medical Devices, Peking University School and Hospital of Stomatology, Beijing, China; 2 Department of Periodontology, Shanghai Stomatological Hospital, Fudan University, Shanghai, China; 3 Department of Stomatology, Peking University Third Hospital, Beijing, China

**Keywords:** aggressive periodontitis, dental implant, longitudinal study, microbiota, residual teeth

## Abstract

**Introduction:**

This study aimed to investigate the evolution of the submucosal microbiota and compare the submucosal and subgingival microbiota in patients with aggressive periodontitis.

**Methods:**

Patients with aggressive periodontitis underwent initial periodontal and surgical therapy, followed by implant restoration. Samples were longitudinally collected from the teeth and implants before the initial therapy (V0), before implantation (V1), one month after abutment connection (V2), one month (V3) and one year (V4) after restoration. The 16S rRNA V3-V4 region of subgingival and submucosal samples was analyzed using the MiSeq platform.

**Results:**

A total of 108 subgingival pooled samples and 24 submucosal pooled samples from 6 patients were sequenced to generate 3,129 operational taxonomic units (OTUs) capturing 12 phyla, 27 classes, 49 orders, 92 families, 195 genera, and 554 species. A considerable microbial shift was observed from 1 month after abutment connection to 1 month after loading (V2 to V3); however, this shift slowed during the remaining study period. One year after loading, a similarity of 76.14% was observed between the microbiota of the implants and that of the teeth.

**Conclusions:**

The submucosal microbiota showed 76.14% similarity in bacterial composition with residual teeth but exhibited more dynamic evolutionary characteristics during the first year after restoration.

## 1 Introduction

Implant-supported restorations are the most widely used method in prosthetic dentistry. A recent meta-analysis revealed that approximately two in three adults with dental implants have peri-implant mucositis, and one in four have peri-implantitis, representing a significant and growing public health challenge (1). Periodontitis is the most common cause of tooth loss and can also increase the risk of peri-implant disease, even when it is well controlled (2–4). Such risk is particularly pronounced in patients with aggressive periodontitis (AgP) (5), a condition marked by rapid periodontal breakdown in systemically healthy young individuals, driven by an exaggerated host response and a more virulent subgingival microbiota (6, 7). In periodontal health, a diverse oral microbiome maintains homeostasis through antimicrobial activity, mucosal barrier reinforcement, and modulation of inflammation (8). Conversely, AgP-associated subgingival communities are enriched in pathogens such as *Aggregatibacter actinomycetemcomitans*, *Porphyromonas gingivalis*, and *Prevotella intermedia*, which form a more interconnected and pathogenic network than in chronic periodontitis (7). These pathogens excessively activate the secretion of cytokines, chemokines, prostaglandins and proteases, and inhibit the local immune response and facilitate periodontal tissue destruction (8, 9). A meta-analysis by Monjeet al. demonstrated that while implant survival rates in AgP patients are comparable to those in periodontally healthy patients, the risk ratio for implant failure is approximately 4.0, underscoring the heightened susceptibility of this population to biological complications (5).

The implant surface provides a solid substrate, similar to natural teeth, for bacterial colonization and biofilm formation. Once implant-supported components are exposed, bacterial attachment occurs, and a complex biofilm can develop within weeks (10–12). Traditional culture-based longitudinal studies have detected a submucosal microbiota with a lower total bacterial load compared to natural teeth but an increased prevalence of periodontal pathogens over time (13–15). Such an increase was more pronounced in patients with a history of periodontitis and might be associated with the subgingival microbiota of remaining teeth (16–19).

The advent of high-throughput sequencing technologies, such as Illumina MiSeq, has broadened our understanding of the peri-implant microbiome by enabling comprehensive, culture-independent profiling of complex microbial communities at greater depth. Cross-sectional studies using these approaches have revealed that healthy implants harbor a microbiota dominated by facultative anaerobes such as *Streptococcus* and *Neisseria*, with a simpler and less diverse structure compared to teeth. In contrast, peri-implantitis sites are characterized by a denser, more diverse microbial community enriched in pathogens including *P. gingivalis*, *Tannerella forsythia*, *Treponema denticola*, and *Fusobacterium nucleatum* (20, 21).

Longitudinal studies have shown that over time, the submucosal microbiota undergoes a compositional shift from early commensal colonizers to a complex community dominated by periodontal pathogens, concomitant with a gradual increase in microbial richness and phylogenetic diversity (22). In patients with a history of periodontitis, this shift appears accelerated, with the genera *Porphyromonas*, *Tannerella*, *Treponema*, and *Prevotella* increasing while health-associated genera such as *Streptococcus* and *Actinomyces* decrease over time (23). Several factors may drive this pathogenic shift. The establishment of the peri-implant microbiome is governed by pioneer species that create a niche favoring the recruitment of phylogenetically related taxa, thereby shaping the trajectory of community succession (24). Meanwhile, adjacent residual teeth may act as a persistent microbial reservoir, from which periodontal pathogens may continuously translocate to the peri-implant sulcus, further aggravating the microbial dysbiosis (16, 17, 25).

Given the heightened susceptibility of AgP patients to biological complications and the limited longitudinal data on their submucosal microbiota, this study longitudinally observed the short-term and long-term shifts in the submucosal microbiota during the first year after implant restoration, and further compared these microbial profiles with those of residual teeth.

## 2 Materials and methods

The clinical trial was registered in ClinicalTrials.gov with registration number NCT03090282 (24/03/2017). The protocol of this longitudinal study was approved by the Ethics Committee of Peking University School and Hospital of Stomatology (PKUSSIRB-201525102). All participants signed informed consent forms. Given the exploratory nature of this longitudinal study, a formal power calculation was not performed. The sample size of six participants was primarily determined based on practical considerations and with reference to previous similar longitudinal microbiome studies in AgP patients.

### 2.1 Participants

Subjects were included in this study if they fulfilled the following criteria:

1. Age 18–35 years,
2. aggressive periodontitis (26), and
3. presence of hopeless teeth that need to be extracted, or being partially edentulous, requiring a single-tooth implant in the posterior region. Staging and grading were performed, and all patients exhibited Stage III–IV Grade C periodontitis (27). Patients with a history of scaling and root planning (SRP) or antibiotic therapy within the last six months were excluded. Participants who were pregnant, lactating, or smokers were also excluded.

The participants received oral hygiene instructions, supragingival scaling, and full-mouth periapical radiography one week before baseline. At baseline (V0), a full-mouth examination was performed, including probing depth (PD), attachment loss (AL), and bleeding index (BI). Subsequently, the participants underwent scaling and root planing (SRP) and were re-evaluated six weeks later. Residual periodontal pockets were treated using re-SRP or periodontal surgery. Six months later, cone-beam computed tomography was performed to evaluate crestal bone morphology and dimensions (V1). All patients underwent a two-stage surgical procedure performed by the same periodontist (YXQ). After local anesthesia and flap elevation, tapped-in, locking-tapered titanium surface implants (Bicon, Dental Implant, Boston, MA, USA) measuring 5.0 mm in width and 8.0 mm in length were allocated 2–3 mm below the crest of the bone, following the manufacturer's instructions. Finally, the flaps were sutured, and primary closure was performed. Antibiotic therapy (amoxicillin, 250 mg three times daily for seven days) and chlorhexidine mouth rinse (10 mL twice daily for 14 days) were administered after surgery. The sutures were removed 14 days after surgery. Three months after the first stage of surgery, a healing abutment (5 mm × 6.5 mm) was connected. One month after the abutment connection, an implant-level impression was taken (V2), and a cement-retained zirconia crown was fabricated. The crowns and abutments were cemented extraorally. Clinical and radiographic evaluations of the implants were performed one month (V3) and one year (V4) after restoration. The probing pocket depth (PPD) was assessed at six sites, and the modified BI (mBI) was assessed at two sites. Patients underwent routine maintenance visits during the entire process at 3- to 6-month intervals. The kappa values were 0.897 for PD and 0.780 for AL.

### 2.2 Sampling and DNA sequencing

Detailed information on the sampling and sequencing procedures has been reported in previous studies (28). Before SRP, four pooled subgingival plaques were sampled from the posterior mesial (L1: teeth #3, #4, #19, and #20), posterior buccal (L2: teeth #13, #14, #29, and #30), anterior mesial (L3: teeth #7, #8, #23, and #24), and anterior buccal (L4: teeth #9, #10, #25, and #26) sites with PD ranging from 3 mm to 7 mm in each patient. Subgingival samples from the same site were obtained at each follow-up visit (TV0-TV4). Submucosal samples were also taken from the mesial and buccal aspect at visits V2 to V4 (IV2, IV3, and IV4). When two submucosal samples were taken from one participant, they were labelled F1 and F2, respectively, according to the order in the FDI World Dental Federation notation system. The flow of the study is shown in Supplementary Figure S1 (Additional Supplementary information may be found in the Supplementary material section).

Before sampling, the supragingival or supramucosal plaque was removed, and the sampling sites were isolated using cotton rolls. Subgingival or submucosal samples were collected by inserting filter papers (2 × 10 mm, Whatman Grade 3MM Chr; Whatman International Ltd., Maidstone, UK) followed by curette sampling using a Gracey curette for teeth or plastic curette for implants. First, a filter paper was inserted into the sampling sites for 30 s. Then, a curette was used to obtain plaques at the same site. Filter paper and curette samples from the same location of one participant were merged into a sterile 1.5 mL microcentrifuge tube (Axygen®, Corning, NY, USA). Samples visibly contaminated with blood were discarded at the time of collection and resampled if conditions permitted. Immediately after collection, 200 μL of sterile phosphate-buffered saline (PBS, pH 7.4, Gibco®, Thermo Fisher Scientific, MA, USA) was added to each tube, followed by vigorous shaking for 1 h. The samples were then centrifuged, and the resulting bacterial pellets were separated and stored at −80 °C until further DNA extraction. All samples were transported under strict cold-chain conditions.

DNA was isolated using the QIAamp DNA Mini Kit (Qiagen, Hilden, Germany). The amount and quality of the DNA were determined using a Nanodrop2000 (Thermo Fisher Scientific, MA, USA). The 16S rRNA gene V3-V4 hypervariable region was amplified with primers 338F (5’-GTACTCCTACGGGAGGCAGCA-3’) and 806R (5’-GTGGACTACHVGGGTWTCTAAT-3’). The amplification procedures were as follows: one cycle 94 °C for 5 min, 30 cycles 95 °C for 30 s, 56 °C for 30s, 72 °C for 40 s. and a final cycle of 72 °C for 10 min. The 16S rRNA gene V3-V4 hypervariable region was sequenced on the MiSeq platform at Allwegene Company. The sequence data were submitted to the GenBank database under the accession number PRJNA379905.

### 2.3 Statistical analysis

High-quality reads were clustered into operational taxonomic units (OTUs) with 97% similarity. The Ribosomal Database Project Classifier tool was used to classify all the sequences into different taxonomic groups against the Human Oral Microbiome Database (15.22). The generated sequences were analyzed using the Quantitative Insights into Microbial Ecology package (29) and Mothur. The variation in submucosal microbiota within one year of restoration was divided into two parts by three time points: 1 month after abutment connection to 1 month after loading (V2 to V3, named short-term phase) and 1 to 12 months after restoration (V3 to V4, named long-term phase). Alpha diversity indices were compared between groups or parts using the Mann–Whitney U or Kruskal–Wallis test. Analysis of the similarities (ANOSIM) was conducted to compare the intra- and inter-group similarities (30). Beta diversity comparisons (Jaccard index and weighted UniFrac distance) were performed only in the 4 patients who completed all visits, and were compared using either student-t test or one-way ANOVA. Multiple comparisons were performed using p.adjust package with Benjamini-Hochberg method in R version 3.3.2 (R Foundation for Statistical Computing, Vienna, Austria). Metastats was used to detect differentially abundant bacteria between groups in all 6 patients (31). Gene functions predictions were identified according to Phylogenetic Investigation of Communities by Reconstruction of Unobserved States (PICRUSt) based on the Kyoto Encyclopedia of Genes and Genomes (KEGG) database (32). Clinical parameters were compared using the Kruskal–Wallis test or Student's t-test depending on the results of the normality tests.

## 3 Results

Recruitment of participants began in April 2016, and follow-up visits were completed by the end of July 2019. A total of 6 patients (10 implants) with a mean age of 30.5 were enrolled in this study, of which 4 completed all follow-up visits. The clinical characteristics of the participants at each visit are presented in Table 1. Before periodontal treatment, the mean probing depth of the natural teeth was 4.70 mm, which decreased to 2.86 mm before implantation. The median BI changed from 3.23 to 1.80. These clinical characteristics were maintained at low levels throughout the process. Five of the 10 implants were located in the maxillary region with 4 in the molars and 1 in the premolar. The other 5 were located in the molar of the mandible. None of the implants showed clinical signs of biological complications at one-year follow-up. The PPD was 3.29 mm at the end of the study period.

**Table 1:** The clinical parameters of natural teeth and implants.

Visit	V0	V1	V2	V3	V4
Participants	6	6	6	4	5
Gender (M/F)	3/3	3/3	3/3	2/2	3/2
Residual teeth					
PD (full mouth, mm)	4.70 ± 0.62	2.86 ± 0.20	2.58 ± 0.33	2.71 ± 0.31	2.86 ± 0.38
ABI (full mouth)	3.23 (2.57, 3.95)	1.80 (1.63, 2.35)	1.46 (1.09, 2.02)	1.30 (1.09, 1.63)	1.43 (0.88, 1.88)
PD (sampling sites, mm)	4.63 ± 1.49	3.36 ± 1.02	2.44 ± 0.71	2.22 ± 0.72	2.22 ± 0.77
AL (sampling sites, mm)	4.72 ± 1.56	3.92 ± 1.26	3.59 ± 1.05	3.68 ± 1.01	3.67 ± 1.18
BI (sampling sites)	3.0 (2.125, 4.0)	2.00 (2.00,2.50)	1.75 (1.00,2.00)	1.75 (1.50,2.00)	1.00 (1.00,1.50)
Implants					
Number			10	6	8
PPD (mm)			-	3.00 ± 0.57	3.29 ± 0.79
mBI			-	1.50 (1.00, 1.625)	1.50 (1.50, 2.00)

BI, bleeding index; PD, probing depth; AL, attachment loss.

Values are the mean ± standard deviations or median value (inter-quartile range).

*Mann–Whitney U test for BI and paired t test for PD and AL.

A total of 132 pooled samples were sequenced, including 24 submucosal and 108 subgingival samples. The total number of sequences generated was 8,655,896, with a mean number of 65,575, generating 3,129 OTUs and capturing 12 phyla, 27 classes, 49 orders, 92 families, 195 genera, and 554 species.

### 3.1 Diversity and microbiota structure

Alpha diversity, presented by Chao1, phylogenetic diversity whole tree, Shannon, and Simpson indices, showed no significant difference between subgingival samples and submucosal samples during the implantation process (TV2 *vs*. IV2, TV3 *vs*. IV3, and TV4 *vs*. IV4) or among submucosal samples (IV2-IV4) (Figure 1, detailed data in Supplementary Table S1). Principal coordinate analysis (PCoA) was performed based on the Weighted UniFrac distance matrix to depict the microbial community structure of submucosal and subgingival samples (Figure 2). Longitudinally, ANOSIM showed significant differences and weak separation between groups IV2 and IV4 (R^2^ = 0.15, *P* = 0.034, ANOSIM), an insignificant difference between groups IV2 and IV3 (R^2^ = 0.05, *P* = 0.215, ANOSIM), or between groups IV3 and IV4 (R^2^ = 0.14, *P* = 0.067, ANOSIM). Significant differences but very weak separations were observed between the natural teeth and implants one month and one year after restoration (V3: R^2^ = 0.16, *P* = 0.040; V4: R^2^ = 0.17, *P* = 0.028, ANOSIM). No significant difference was observed between the implants and teeth one month after abutment connection (R^2^ = 0.11, *P* = 0.056, ANOSIM).

**Figure 1:**
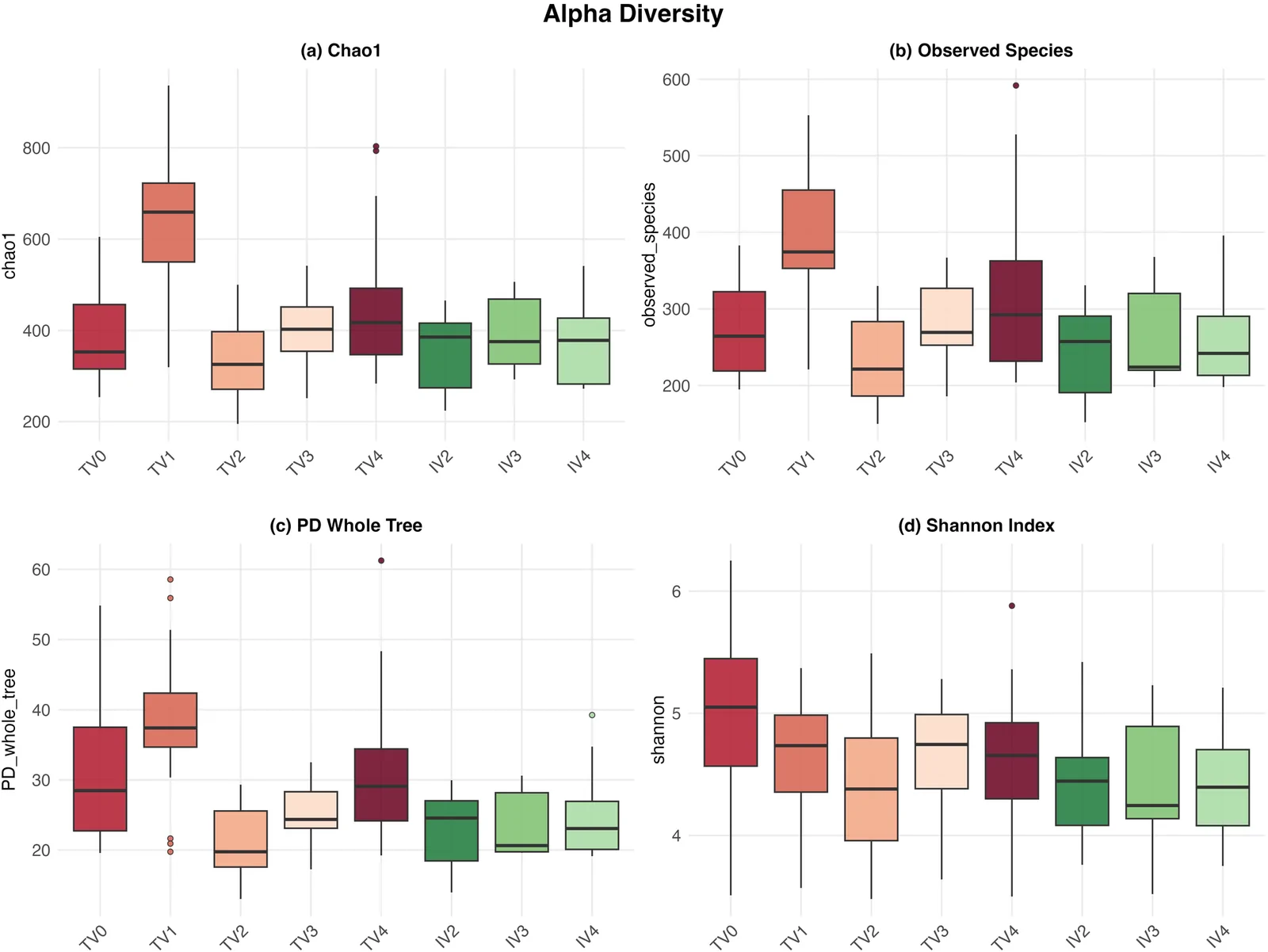
Alpha diversity of subgingival and submucosal microbiota across time points. **(a)** Chao1, **(b)** observed species, **(c)** PD whole tree, and **(d)** Shannon index. Sample groups: TV0-TV4 (Teeth, time points V0-V4) and IV2-IV4 (Implants, time points V2-V4). Time points are defined as follows: V0, baseline; V1, before implantation; V2, one month after abutment connection; V3, one month after loading; V4, one year after loading.

**Figure 2:**
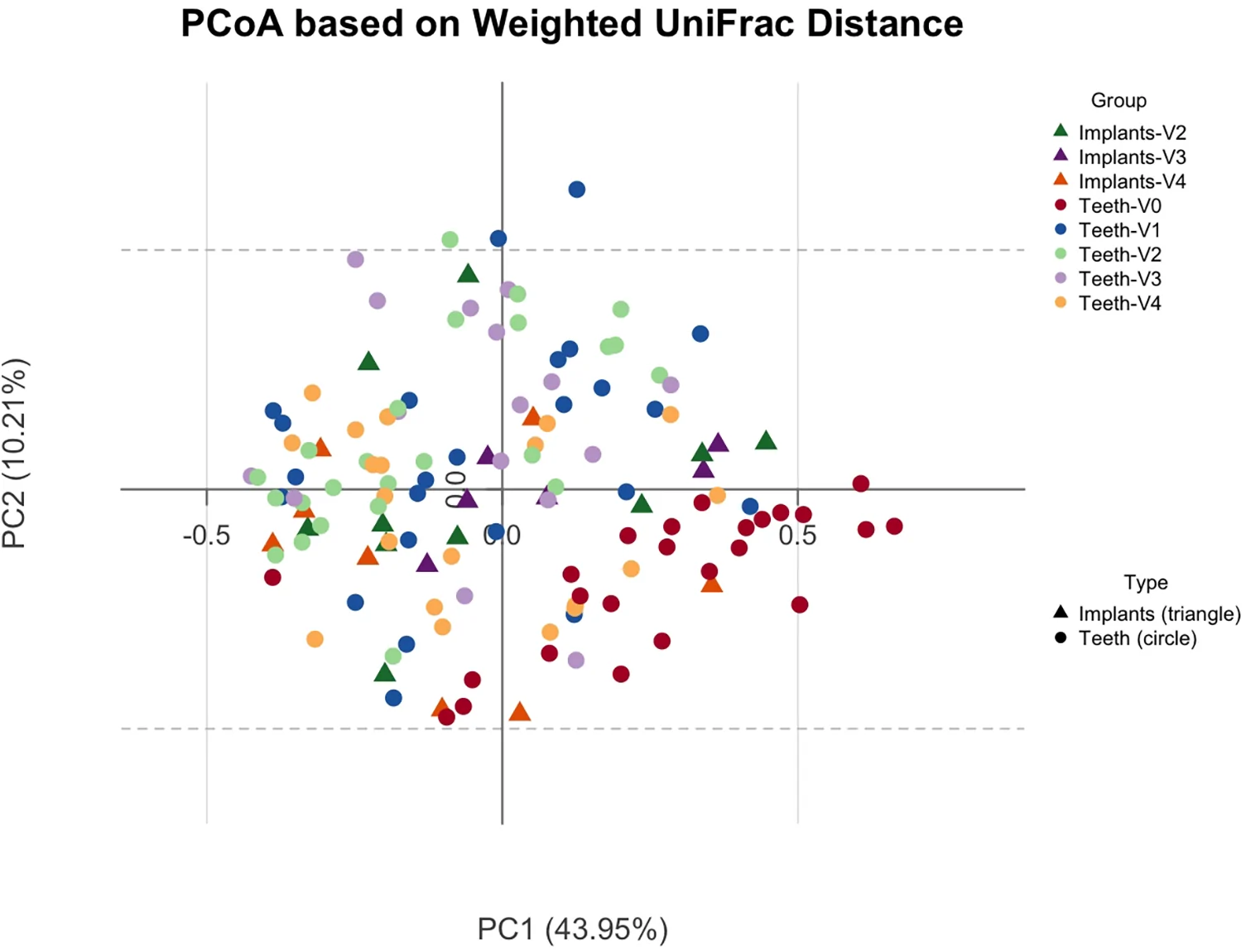
Principal coordinate analysis (PCoA) plots based on weighted uniFrac distance metrics. Each point represents an individual sample: circles represent teeth; triangles represent implants. Different colors indicate different time points (V0–V4) and sample type (teeth/implants). Time points are defined as follows: V0, baseline; V1, before implantation; V2, one month after abutment connection; V3, one month after loading; V4, one year after loading.

The Jaccard index is a statistical method defined as the ratio of shared species to the total union of species between two samples, considering only presence/absence rather than abundance. For each patient, we calculated this index between the implant sample and the four natural tooth samples at each time point, and then compared the values across visits. This value was low at V2 (genus-level: 68.71%, species-level: 46.38%), increased at V3 (genus-level: 74.17%, FDR *p* = 0.001; species-level: 55.54%, FDR *p* = 0.003, paired student-t test), and remained high until V4 (genus-level: 76.14%; species-level:51.83%). The Weighted UniFrac Distance considers not only the presence or absence of species but also the abundance of species. This value was low at V2 (0.43), increased to 0.53 at V3, then decreased to 0.39 at V4 (V3-V4, FDR *p* = 0.01) (Table 2).

**Table 2:** Comparison of diversity between implants and teeth among different timepoints (values are the mean ± standard deviations, compared by one-way ANOVA followed by *post-hoc* LSD, *p* values were adjusted for the FDR correction).

Group	Jaccard Index (genus level)			Weighted UniFrac Distance		
	IV2	IV3	IV4	IV2	IV3	IV4
TV1	0.66 ± 0.06	0.67 ± 0.06	0.71 ± 0.07	0.51 ± 0.12	0.53 ± 0.11	0.44 ± 0.10
TV2	0.69 ± 0.06	0.70 ± 0.05	0.67 ± 0.08	0.43 ± 0.15	0.49 ± 0.16	0.38 ± 0.12
TV3	0.67 ± 0.07	0.74 ± 0.05	0.72 ± 0.06	0.47 ± 0.13	0.53 ± 0.12	0.42 ± 0.13
TV4	0.69 ± 0.07	0.70 ± 0.08	0.76 ± 0.10	0.49 ± 0.13	0.50 ± 0.13	0.39 ± 0.10
FDR P	0.377	0.0015	0.0015	0.459	0.573	0.459

The microbiota of the implants and natural teeth was dynamic. The Jaccard index between the implants and residual teeth at the same time point was higher than that between the teeth at other time points especially in V3 and V4 (V3, FDR *p* = 0.0015; V4, FDR *p* = 0.0015, ANOVA). The Weighted UniFrac Distance results did not show this phenomenon between the implants and teeth (Table 2).

Microbial shifts were compared between the short- and long-term phases (Table 3). The Jaccard index at the genus level showed that the similarity in bacterial composition was lower in the short-term phase than in the long-term phase, with a mean difference of 0.03 (Cohen's d = 0.50, FDR *p* = 0.045). This marginally significant difference suggests a potential distinction between the two developmental phases, indicating greater variation of submucosal bacteria in the short-term phase than in the long-term phase. The changes in the Weighted UniFrac Distance over the short- and long-term phases showed no significant differences. Alterations in the plaque structure around the implants were greater than those around the teeth over the long-term phase (*P* = 0.027, student-t test).

**Table 3:** Comparison of diversity in two phases (values are the mean ± standard deviations, student-t test).

Parameters	Sample Type	V2-V3	V3-V4	FDR P
Jaccard Index (Genus-level)	Natural Teeth	0.70 ± 0.05	0.71 ± 0.06	0.450
	Implants	0.68 ± 0.06	0.71 ± 0.06	0.045
	FDR P	0.345	0.736	
Weighted UniFrac Distance	Natural Teeth	0.39 ± 0.14	0.45 ± 0.11	0.099
	Implants	0.47 ± 0.18	0.58 ± 0.15	0.204
	FDR P	0.207	0.027	

Intra and interindividual differences were also considered. A comparison of the similarity in bacterial composition between dental implants and residual teeth and between dental implants in different individuals was calculated (Table 4). The Jaccard similarity between implants and natural teeth was 0.75 ± 0.09, while the similarity between implants from different individuals was 0.68 ± 0.06. The mean difference was 0.07 (95% CI: 0.03 to 0.11), with a Cohen's d of 0.92, indicating a large effect size. This suggests that patient-specific factors have a substantial influence on microbiota similarity, beyond the local differences between implant and tooth surfaces.

**Table 4:** Comparison of inter-individual and intra-individual differences (values are the mean ± standard deviations, student-t test, *p* values were adjusted for the FDR correction).

Parameters	Comparison	V2	V3	V4
Jaccard Index	With other implants	0.62 ± 0.07	0.72 ± 0.039	0.68 ± 0.06
	With natural teeth	0.70 ± 0.06	0.74 ± 0.045	0.75 ± 0.09
	FDR P	<0.001	0.138	0.0015
Weighted UniFrac Distance	With other implants	0.49 ± 0.14	0.49 ± 0.12	0.47 ± 0.15
	With natural teeth	0.41 ± 0.17	0.49 ± 0.14	0.39 ± 0.16
	FDR P	0.048	0.933	0.093

### 3.2 Bacterial composition

The most abundant phyla, *Firmicutes*, *Proteobacteria*, *Bacteroidetes*, *Fusobacteria*, and *Actinobacteria* accounted for 95.45% of all bacteria (Supplementary Table S2). The top 10 genera of each sample accounted for an average of 85.04% of the total abundance, with the highest being 98.03% and the lowest being 66.13%. Most of these bacteria belonged to *Streptococcus*, *Fusobacterium*, *Neisseria*, *Haemophilus*, and *Capnocytophaga*. Other bacteria (more than 100 genera) were detected at very low abundances. The distribution of the top 25 genera in each sample is shown in Figure 3. We therefore focused on the most abundant genera to track their longitudinal changes (Figure 4). Some differences were observed before FDR correction (as indicated in the figure); however, none of them remained statistically significant after FDR correction (adjusted *p* > 0.05).

**Figure 3:**
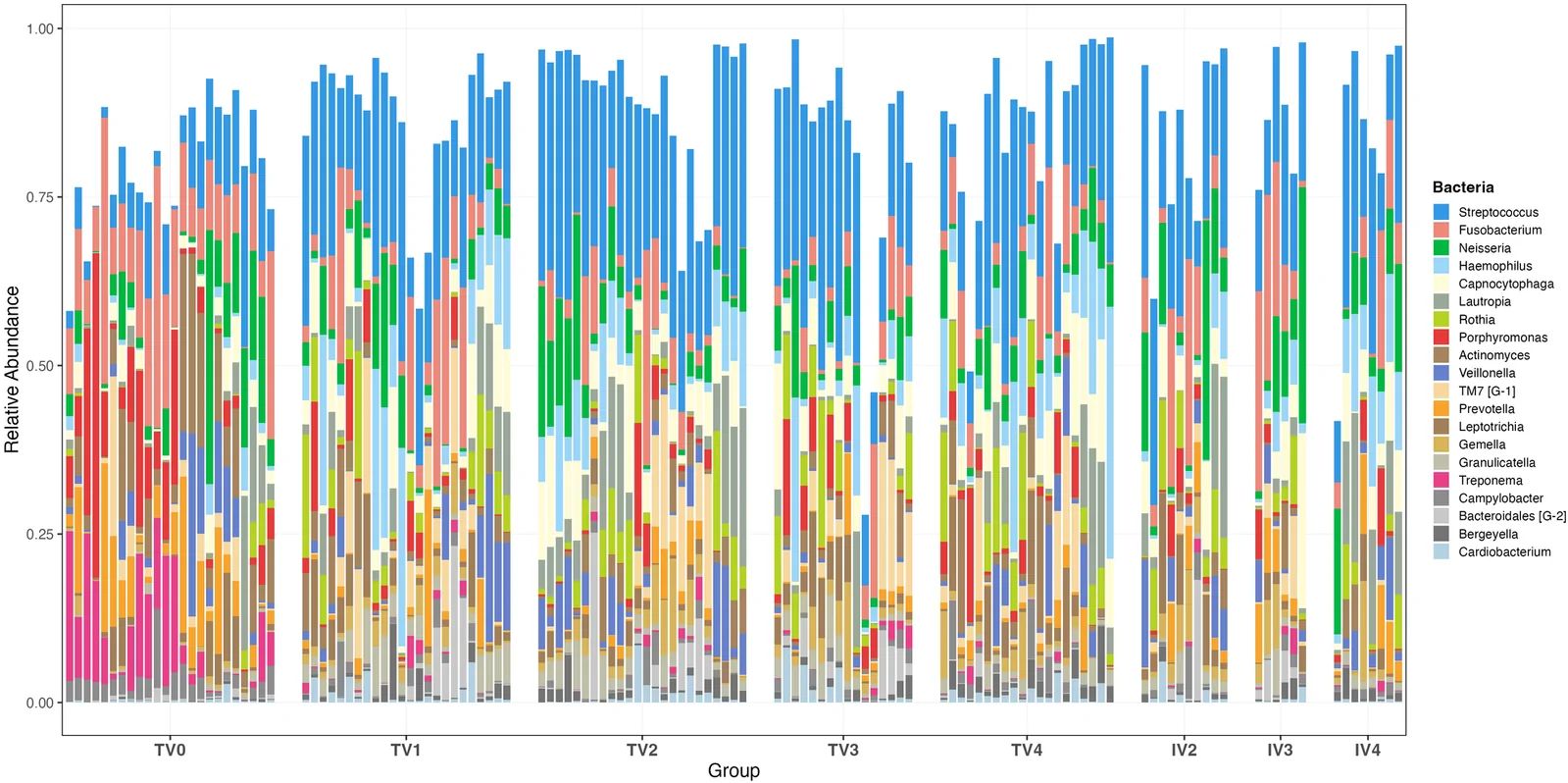
Distribution of top 20 bacterial genera in subgingival and submucosal microbiota. Stacked bar plot showing the relative abundance of bacterial genera in each biological replicate. Sample groups: TV0-TV4 (Teeth, time points V0-V4) and IV2-IV4 (Implants, time points V2-V4). Time points are defined as follows: V0, baseline; V1, before implantation; V2, one month after abutment connection; V3, one month after loading; V4, one year after loading. Each vertical bar represents one biological replicate.

**Figure 4:**
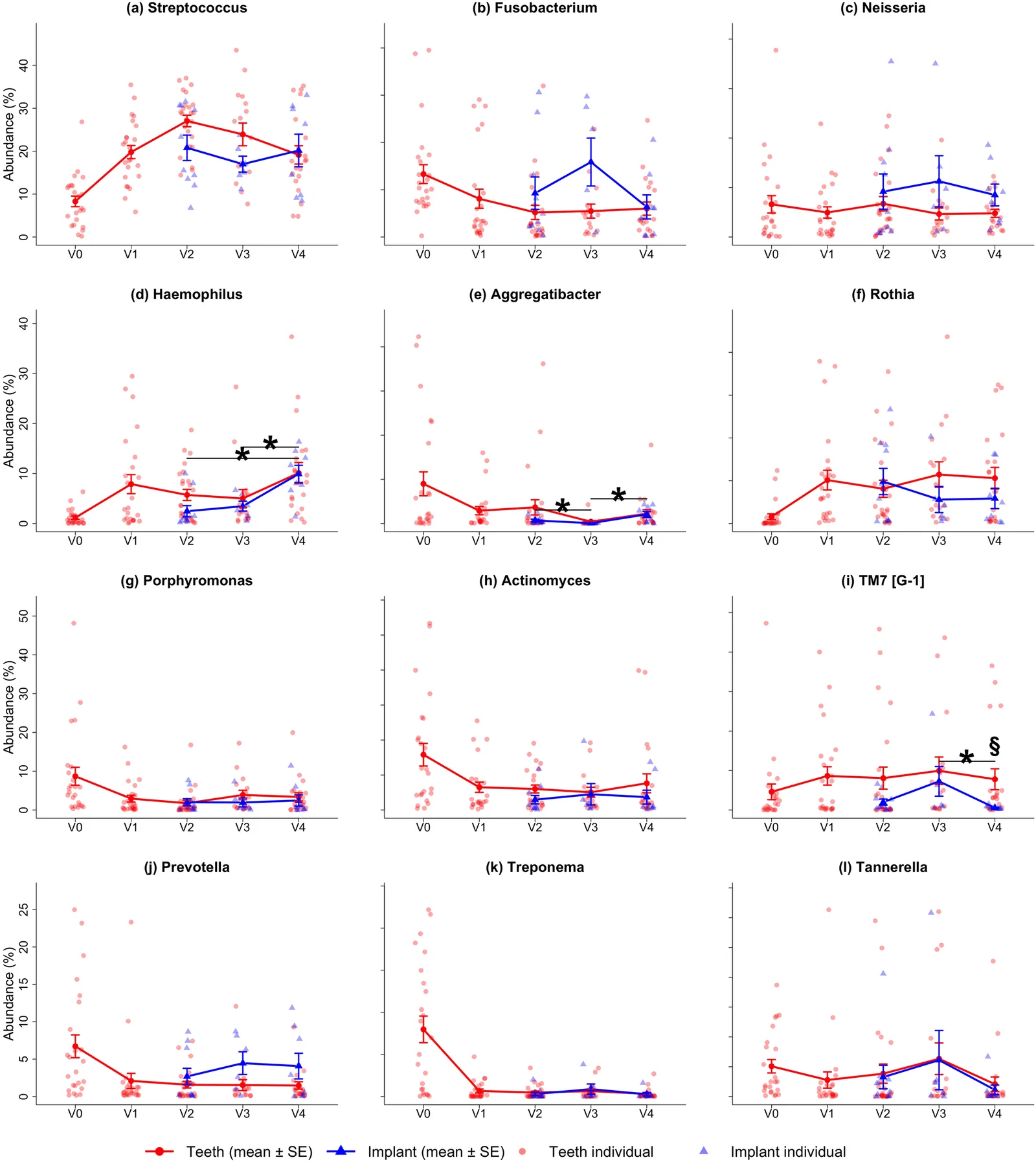
The longitudinal changes of bacteria in submucosal microbiota and a comparison of bacteria between teeth and implants. **(a)** *Streptococcus*, **(b)** *Fusobacterium*, **(c)** *Neisseria*, **(d)** *Haemophilus*, **(e)** *Aggregatibacter*, **(f)** *Rothia*, **(g)** *Porphyromonas*, **(h)** *Actinomyces*, **(i)** *TM7 [G-1*], **(j)** *Prevotella*, **(k)** *Treponema*, **(l)** *Tannerella*. V0, baseline; V1, before implantation; V2, one month after abutment connection; V3, one month after loading; V4, one year after loading. §*p* < 0.05 for comparisons between implants and teeth; *p < 0.05 for comparisons between two time points within submucosal microbiota (Metastats). All *p*-values shown are uncorrected; none remained statistically significant after FDR adjustment (adjusted *p* > 0.05).

Prior to periodontal therapy, obligate anaerobes such as *Porphyromonas* and *Treponema* were present at relatively high abundances in teeth. Following treatment (V1), their abundance declined substantially and remained low throughout the study period. For instance, *Treponema* decreased from 7.97% at V0 to 0.66% at V1, and stayed at similarly low levels in both niches from V2 to V4 (0.26%–0.87%), with no implant-specific enrichment. One month after abutment connection, the implant-associated microbiota was predominated by *Streptococcus* (20.79%), *Fusobacterium* (9.27%), *Neisseria* (9.17%), *Lautropia* (5.39%), and *Capnocytophaga* (5.06%). *Streptococcus* showed the highest abundance (16.98%–20.79%) from V1-V4. The abundances of *Haemophilus* gradually increased from V2 to V4 (V2-V4*, p* = 0.003, Metastats). In contrast, *Prevotella* showed a non-significant trend toward increase during the same period (FDR-adjusted *p* > 0.05). The genera *Neisseria* and *Rothia* showed a non-significant decreasing trend during follow-up. The abundances of *TM7* increased from V2 to V3 and decreased at V4, while *Fusobacterium* showed the same tendency. The abundance of *Aggregatibacter* showed a different trend, declining from V2 to V3 and subsequently rising from V3 to V4. At V4, the peri-implant microbiota was predominated by *Streptococcus* (20.17%), *Haemophilus* (9.91%), *Lautropia* (9.90%), *Neisseria* (8.49%), *Fusobacterium* (6.36%), and *Capnocytophaga* (6.01%), together representing 60.84% of the total community.

Comparisons between subgingival and submucosal microbiota were also performed at corresponding time points. One month after abutment exposure, the relative abundance of *Haemophilus* and *Bergeyella* around the implants was lower than that around the natural teeth, while the abundance of *Fusobacterium*, *Rothia*, and *Neisseria* were comparatively higher around the implants than around the natural teeth. One month after restoration, the relative abundances of *Fusobacterium*, *Neisseria*, *Veillonella* and *Prevotella*, in implants were higher than those in natural teeth. However, the relative abundances of *Streptococcus*, *Rothia*, *TM7 [G-1]*, *Corynebacterium*, and *Porphyromonas* were lower than those in the natural teeth. The abundances of *TM7 [G-1]* and *Cardiobacterium* were lower in submucosal samples than in subgingival samples one year after restoration, while those of *Neisseria* and *Prevotella* were insignificantly higher in submucosal samples (Figure 4).

### 3.3 Functional pathways prediction

To predict the differences in functional pathways of the submucosal microbiome, we analyzed signaling pathways with a relative abundance >1% between adjacent follow-up time points. The results revealed a significant functional remodeling of the predicted metagenome from V3 to V4, with a total of 15 pathways-involving DNA replication, ribosome synthesis, amino acid metabolism, and antibiotic biosynthesis-showing a marked decrease in relative abundance (FDR *p* < 0.05), suggesting a possible reduction in the predicted functional potential for growth and replication, which may reflect a transition toward a more stable community structure (Supplementary Table S3). In contrast, no significant functional changes were observed in the implant-associated microbiome between V2 and V3 (Supplementary Table S4).

There were also differences in pathways between implants and natural teeth. One month after restoration, compared with natural teeth, implant samples were enriched in pathways related to cell growth and defense (although the FDR-adjusted *p*-values for some pathways approached 0.05) (Supplementary Table S5). However, by V4, the functional differences between implants and natural teeth had almost completely disappeared (FDR *p* > 0.05) (Supplementary Table S6).

## 4 Discussion

Periodontitis patients are more susceptible to implant failure and greater bone loss as compared with periodontally healthy individuals (4, 33). This risk is more pronounced in patients with aggressive periodontitis (2, 5, 34). In the present study, we longitudinally observed submucosal microbiota in patients with aggressive periodontitis and found that it shared 76.14% genus-level similarity with the subgingival microbiota, yet exhibited microbial structural differences from that around teeth.

Similarities in microbial profiles between implants and residual teeth have been confirmed in numerous studies using different microbiological techniques (12, 16, 35). Freitas et al. compared the bacterial composition of implants and contralateral teeth in periodontal healthy individuals and found a similarity of 62% one year after restoration (25). As shown in studies based on traditional microbiological methods, the microbiota around implants was associated with adjacent teeth as well as contralateral occlusal teeth (13, 36, 37); hence, to comprehensively depict the influence of residual teeth on the submucosal microbiota, samples from multiple locations were chosen in the present study. Our study showed that 1 month after abutment exposure, the similarity between the submucosal and subgingival bacterial composition was 68.71%. This similarity increased to 76.14% after one year of functional loading. Moreover, the similarity between implant and residual tooth microbiota was highest when compared at the same time point than across different time points. This similarity may also be explained by alternative factors, such as the shared oral environment, salivary flow, and dietary habits, which collectively influence the microbial communities colonizing both teeth and implants. Weighted UniFrac distances showed a non-linear trend over time. This trend suggests that incorporating abundance and phylogenetic information reveals divergence between implant and tooth microbiota over time. Similar results were observed in species composition analysis. Most bacteria in the peri-implant microbiota were similar to those in natural teeth during the one-year follow-up period. However, an elevated abundance of the genera *Prevotella* and *Neisseria* was found in implants compared to teeth (approximately two times higher in implants than in teeth). Previous studies have also shown a higher abundance of *Neisseria* and *Prevotella* on titanium implant surfaces than on natural teeth or zirconia surfaces (3, 38, 39). The higher abundance of *Prevotella* and *Neisseria* on implants may be attributed to titanium surface properties that modulate salivary protein adsorption and bacterial colonization (25, 40). *Prevotella*, a predominant submucosal genus comprising periodontal pathogen *Prevotella intermedia* and *Prevotella nigrescens* (41–43), invades epithelial cells and exhibits higher affinity for titanium (44–46). While cross-sectional studies have reported its enrichment at peri-implantitis sites (47–49) and its role as a pathobiont in periodontitis, the relationship between *Prevotella* and implant diseases remains unclear (8).

In the present study, obligate anaerobes such as *Porphyromonas* and *Treponema* showed a marked reduction in relative abundance following periodontal therapy, while *Streptococcus* emerged as the dominant genus, accompanied by significant improvements in clinical parameters. This pattern aligns with Krajewski et al., who reported that non-surgical periodontal therapy significantly reduces red complex bacteria (50, 51). As supportive periodontal care significantly enhances the reduction of *P. gingivalis* and *T. denticola* at 6 months after therapy, the sustained low abundance of these pathogens throughout the follow-up period in our AgP cohort likely reflects the supportive therapy rigorously implemented in this high-risk population (50). These findings highlight the importance of effective periodontal therapy and sustained maintenance in preserving a health-compatible microbiome throughout the implant treatment course in periodontally susceptible patients.

Vílchez et al. conducted a longitudinal study and analyzed submucosal samples at 1 week, 4 weeks, and 3 years post-implantation (22). The study reported early biofilms dominated by facultative anaerobes such as *Haemophilus parainfluenzae* and *Neisseria elongata*, which gradually declined, while anaerobic/proteolytic taxa including *Prevotella*, *P. gingivalis*, and *F. nucleatum* became significantly enriched at 3 years. Consistent with their findings, the present study also observed the persistence of early colonizers—such as *Streptococcus*, *Neisseria*, and *Haemophilus*—at high relative abundances throughout the 1-year follow-up, supporting the foundational role of these taxa in early biofilm development around implants (24). However, in contrast to Vílchez et al., we did not observe a significant increase in anaerobic pathogens at 1 year. This discrepancy may be partly attributable to the platform-switching design of the implants used in the present study, which reduces microleakage at the implant-abutment interface and may delay the ingress of periodontal pathogens (52). Alternatively, the 1-year follow-up may fall short of the time point at which pathogenic taxa accumulate, as observed at 3 years by Vílchez et al. Alterations in the emergence profile and the introduction of functional loading also led to distinct temporal patterns in several taxa. The abundance of *TM7[G-1]* and *Fusobacterium* was high one month after abutment exposure and restoration, and its abundance was reduced one year after loading. *TM7* is an organism characterized by an ultrasmall cell with highly reduced genomes and restricted metabolic capacities (53). Like *Fusobacterium*, it can also interact with various bacteria and regulate the structure and function of a microbial community, thus playing an important role in plaque formation (54). It is plausible that the early appearance of *TM7* and *Fusobacterium* creates preconditions for the accumulation of late-colonizing bacteria, potentially facilitating further colonization by periodontal pathogens. The successful cultivation of *TM7x* will facilitate future studies to elucidate the characteristics and mechanisms of *TM7*, thus helping evaluate this hypothesis (55)**.** *Haemophilus* was previously considered related to implant health, with decreased abundance in the presence of suppuration (43). In this study, the relative abundance of *Haemophilus* increased significantly one year after loading.

One year after restoration, the difference between implants in different individuals was greater than that between implants and teeth in the same individual, indicating a high level of intersubject variation. Implants and teeth in the same individual are expected to establish similar biofilms since the oral environment and nutritional sources (saliva) are shared. Previous horizontal studies have found a significant component of interpersonal differences that is more predominant than anatomical niche differences (submucosal or subgingival) (48, 56, 57). This result also suggests that the maturation of submucosal microbiota is mostly driven by the patient, while the special environment and surface offered by implants may have a limited effect one year after loading (42).

There are also characteristics of the evolution of submucosal plaques. By dividing the study period into two parts (one month after abutment connection to one month after restoration; one month to one year after restoration), the bacterial composition changed considerably in the early stage and was relatively stable in the late stage. Previous studies on natural teeth and implants detected specific bacteria within weeks, although building a mature typical biofilm takes at least several months (10, 58–60). There are also several macro changes in the early stages, such as the emergence of fresh peri-implant pockets, shifts in emergence profiles, and functional loading. The use of antibiotics and chlorhexidine may also have affected the evolution of the microbiota. In contrast, the emergence profiles and functional loading remained stable in the late stage. Fewer changes in predicted functional pathways were observed in the early stages, whereas more pathways changed in the late stage. Functional adaption might be slower than bacteria adhesion.

According to the Human Oral Microbiome Database (HOMD), approximately 70% of oral prokaryotes are known to be culturable (61). The key species within the core genera examined in this study—including *Streptococcus*, *Fusobacterium*, *Porphyromonas*, *Neisseria*, and *Haemophilus*—have well-documented culture records, and even the typically fastidious *TM7* has been successfully isolated via co-culture. Although this study primarily presents genus-level results, the main detected taxa possess cultivability potential at the species level. Culture-based methods enable viable bacterial recovery, phenotypic characterization, antimicrobial susceptibility testing, and strain-level identification, which are essential for establishing causal relationships between pathogens and disease. In contrast, 16S rRNA sequencing offers broader and deeper community coverage, detecting fastidious, low-abundance, or uncultivated taxa, making it particularly suitable for revealing ecological dynamics. Since our primary objective was to track longitudinal compositional shifts, the MiSeq platform was selected given its high throughput, broad coverage, cost-effectiveness, and ability to detect rare or fastidious taxa. Future integration of multi-omics and culture-dependent approaches would help verify the viability and functional activity of key taxa and strengthen the biological relevance of sequencing-based findings.

The strength of this study is the longitudinal observation of the whole process of implantation in periodontitis patients, including the longitudinal tracking of submucosal microbiota changes up to 1-year post-restoration and the comparison of microbial profiles between implants and teeth over the same period. A small sample size and patient dropouts reduce statistical power, limit mixed-effects modeling, and raise concerns regarding pseudoreplication and overfitting. Sample pooling may also mask site-specific microbial variability. Therefore, these exploratory findings warrant validation in large-scale, multicenter studies using site-specific sampling and mixed-effects models.

## 5 Conclusions

This longitudinal study elucidates the influence of residual teeth on the maturation of submucosal microbiota, which shows not only overlaps in community composition but also a characteristic high abundance of *Prevotella*, *Neisseria*, and *Haemophilus* in submucosal samples. The evolution of submucosal microbiota showed more changes in microbial structure compared with that of the residual teeth within one year of restoration.

## Data Availability

The datasets presented in this study can be found in online repositories. The names of the repository/repositories and accession number(s) can be found in the article/Supplementary Material.
