# Supplementary material for: The shifts of submucosal microbiota in treated patients with aggressive periodontitis
Source: Front Oral Health. 2026 Sep 22;7:1855289. doi: 10.3389/froh.2026.1855289 (PMC13638358; doi:10.3389/froh.2026.1855289)
Supplement: Supplementary file 1 [file Table1.docx]

**Supp. Table S1: Phylogenetic diversity of subgingival and submucosal samples (Values are the mean ± standard deviations).**

|  |  | Chao1 | Observed species | Phylogenetic diversity whole tree | Shannon | Simpson |
| --- | --- | --- | --- | --- | --- | --- |
| Teeth | V0 | 386.46±90.13 | 274.97±60.78 | 31.51±10.28 | 4.96±0.71 | 0.92±0.06 |
|  | V1 | 638.27±159.72 | 390.38±92.36 | 38.5±9.83 | 4.64±0.51 | 0.91±0.03 |
|  | V2 | 329.61±84.20 | 230.84±55.51 | 21.10±4.70 | 4.39±0.57 | 0.91±0.03 |
|  | V3 | 404.72±87.12 | 279.83±52.05 | 25.35±4.10 | 4.60±0.54 | 0.91±0.03 |
|  | V4 | 457.05±149.80 | 321.40±108.55 | 31.23±10.66 | 4.66±0.56 | 0.92±0.03 |
| Implants | V2 | 351.73±91.54 | 246.25±64.71 | 22.99±5.60 | 4.44±0.50 | 0.90±0.03 |
|  | V3 | 393.25±89.65 | 264.15±75.04 | 23.60±5.42 | 4.41±0.64 | 0.90±0.05 |
|  | V4 | 374.71±96.55 | 264.10±71.60 | 25.40±7.51 | 4.40±0.50 | 0.91±0.03 |
