# Supplementary material for: The shifts of submucosal microbiota in treated patients with aggressive periodontitis
Source: Front Oral Health. 2026 Sep 22;7:1855289. doi: 10.3389/froh.2026.1855289 (PMC13638358; doi:10.3389/froh.2026.1855289)
Supplement: Supplementary file 2 [file Table2.docx]

| Phylum/relative abundance (%) | TV0 | TV1 | TV2 | TV3 | TV4 | IV2 | IV3 | IV4 |
| --- | --- | --- | --- | --- | --- | --- | --- | --- |
| *Firmicutes* | 23.32 | 36.57 | 41.92 | 35.96 | 31.57 | 38.99 | 31.66 | 31.92 |
| *Proteobacteria* | 18.26 | 21.92 | 25.27 | 24.14 | 28.7 | 24.25 | 19.43 | 39.19 |
| *Bacteroidetes* | 20.14 | 15.66 | 14.11 | 15.23 | 15.76 | 14.19 | 21.03 | 14.49 |
| *Fusobacteria* | 16 | 11.27 | 6.81 | 9.27 | 9.55 | 14 | 17.86 | 7.82 |
| *Actinobacteria* | 9.74 | 8.9 | 7.24 | 9.65 | 9.63 | 6.58 | 5.34 | 5.73 |
| *TM7* | 2.58 | 4.54 | 4.14 | 5.01 | 3.95 | 1.38 | 3.68 | 0.28 |
| *Spirochaetes* | 7.97 | 0.66 | 0.48 | 0.64 | 0.31 | 0.3 | 0.87 | 0.26 |
| *Synergistetes* | 1.81 | 0.11 | 0.01 | 0.04 | 0.13 | 0.16 | 0.07 | 0.27 |
| others | 0.18 | 0.37 | 0.02 | 0.06 | 0.4 | 0.15 | 0.06 | 0.04 |

**Supp. Table S2: The relative abundance of phylum in subgingival and submucosal microbiota at different timepoints.**
