# Supplementary material for: The shifts of submucosal microbiota in treated patients with aggressive periodontitis
Source: Front Oral Health. 2026 Sep 22;7:1855289. doi: 10.3389/froh.2026.1855289 (PMC13638358; doi:10.3389/froh.2026.1855289)
Supplement: Supplementary file 3 [file Table3.docx]

**Supp. Table S3: Predicted functional metagenomic changes of the submucosal samples between two follow-ups (V2 *vs*. V3) (relative abundance＞1%).**

| **Level 1** | **Level 2** | **Leve 3** | **IV3** | **IV4** | **P value** | **FDR p** |
| --- | --- | --- | --- | --- | --- | --- |
| Metabolism | Amino acid metabolism  Glycan biosynthesis and metabolism | Biosynthesis of vancomycin group antibiotics | 2.25 | 2.62 | 0.022 | 0.079 |
|  | Metabolism of cofactors and vitamins | Vitamin B6 metabolism | 1.12 | 1.19 | 0.042 | 0.147 |
