# Supplementary material for: The shifts of submucosal microbiota in treated patients with aggressive periodontitis
Source: Front Oral Health. 2026 Sep 22;7:1855289. doi: 10.3389/froh.2026.1855289 (PMC13638358; doi:10.3389/froh.2026.1855289)
Supplement: Supplementary file 4 [file Table4.docx]

**Supp. Table S4: Predicted functional metagenomic changes of the submucosal samples between two follow-ups (V3 *vs*. V4) (relative abundance＞1%).**

| **Level 1** | **Level 2** | **Leve 3** | **IV3** | **IV4** | **P value** | **FDR p** |
| --- | --- | --- | --- | --- | --- | --- |
| Cellular Processes | Cell growth and death | Cell cycle - Caulobacter | 1.65 | 1.51 | 0.013 | 0.024 |
| Genetic Information Processing | Folding, sorting and degradation | Protein export | 1.66 | 1.50 | 0.013 | 0.024 |
|  | Translation | Aminoacyl-tRNA biosynthesis | 1.93 | 1.68 | 0.013 | 0.024 |
|  |  | Ribosome | 1.85 | 1.58 | 0.008 | 0.024 |
|  | Replication and repair | Homologous recombination | 1.78 | 1.52 | 0.020 | 0.030 |
|  |  | Mismatch repair | 1.93 | 1.65 | 0.029 | 0.036 |
|  |  | DNA replication | 1.47 | 1.24 | 0.008 | 0.024 |
| Metabolism | Carbohydrate metabolism | Pentose phosphate pathway | 1.63 | 1.37 | 0.0007 | 0.007 |
|  | Amino acid metabolism | Alanine, aspartate and glutamate metabolism | 1.53 | 1.35 | 0.005 | 0.024 |
|  |  | Lysine biosynthesis | 1.55 | 1.42 | 0.043 | 0.042 |
|  | Metabolism of other amino acids | D-Glutamine and D-glutamate metabolism | 2.52 | 2.12 | 0.003 | 0.019 |
|  |  | D-Alanine metabolism | 2.08 | 1.76 | 0.029 | 0.036 |
|  | Biosynthesis of other secondary metabolites | Streptomycin biosynthesis | 1.65 | 1.30 | 0.0007 | 0.007 |
|  | Metabolism of terpenoids and polyketides | Biosynthesis of ansamycins | 4.02 | 3.05 | 0.008 | 0.024 |
|  |  | Biosynthesis of vancomycin group antibiotics | 2.62 | 2.02 | 0.012 | 0.024 |
|  | Glycan biosynthesis and metabolism | Peptidoglycan biosynthesis | 2.03 | 1.77 | 0.019 | 0.030 |
|  | Energy metabolism | Carbon fixation in photosynthetic organisms | 1.66 | 1.43 | 0.019 | 0.030 |
|  | Nucleotide metabolism | Pyrimidine metabolism | 1.32 | 1.143 | 0.019 | 0.030 |
|  | Lipid metabolism | Synthesis and degradation of ketone bodies | 0.62 | 1.15 | 0.042 | 0.043 |
|  | Metabolism of cofactors and vitamins | One carbon pool by folate | 1.94 | 1.72 | 0.008 | 0.024 |
|  |  | Vitamin B6 metabolism | 1.19 | 1.09 | 0.042 | 0.042 |
