# Supplementary material for: The shifts of submucosal microbiota in treated patients with aggressive periodontitis
Source: Front Oral Health. 2026 Sep 22;7:1855289. doi: 10.3389/froh.2026.1855289 (PMC13638358; doi:10.3389/froh.2026.1855289)
Supplement: Supplementary file 5 [file Table5.docx]

**Supp. Table S5: Predicted functional metagenomic differences between implants and teeth at V3 (relative abundance＞1%).**

| **Level 1** | **Level 2** | **Level 3** | **TV3** | **IV3** | **P value** | **FDR p** |
| --- | --- | --- | --- | --- | --- | --- |
| Metabolism | Metabolism of other amino acids | D-Glutamine and D-glutamate metabolism | 2.27 | 2.52 | 0.049 | 0.056 |
|  | Metabolism of terpenoids and polyketides | Biosynthesis of vancomycin group antibiotics | 2.05 | 2.62 | 0.002 | 0.025 |
|  | Energy metabolism | Carbon fixation in photosynthetic organisms | 1.47 | 1.66 | 0.033 | 0.055 |
|  | Amino acid metabolism | Alanine, aspartate and glutamate metabolism | 1.38 | 1.53 | 0.003 | 0.025 |
|  | Biosynthesis of other secondary metabolites | Streptomycin biosynthesis | 1.43 | 1.65 | 0.040 | 0.056 |
|  | Lipid metabolism | Synthesis and degradation of ketone bodies | 1.11 | 0.62 | 0.027 | 0.055 |
|  | Metabolism of cofactors and vitamins | Biotin metabolism | 1.24 | 1.56 | 0.021 | 0.053 |
|  |  | Vitamin B6 metabolism | 1.10 | 1.19 | 0.013 | 0.050 |
|  | Glycan biosynthesis and metabolism | Peptidoglycan biosynthesis | 1.84 | 2.03 | 0.049 | 0.056 |
|  |  | Lipopolysaccharide biosynthesis | 1.05 | 1.47 | 0.013 | 0.050 |
| Genetic Information Processing | Replication and repair | DNA replication | 1.31 | 1.47 | 0.049 | 0.056 |
|  |  | Mismatch repair | 1.71 | 1.93 | 0.033 | 0.055 |
|  |  | Homologous recombination | 1.55 | 1.78 | 0.021 | 0.053 |
