# Supplementary material for: The shifts of submucosal microbiota in treated patients with aggressive periodontitis
Source: Front Oral Health. 2026 Sep 22;7:1855289. doi: 10.3389/froh.2026.1855289 (PMC13638358; doi:10.3389/froh.2026.1855289)
Supplement: Supplementary file 6 [file Table6.docx]

**Supp. Table S6: Predicted functional metagenomic differences between implants and teeth at V4 (relative abundance＞1%).**

| **Level 1** | **Level 2** | **Level 3** | **TV4** | **IV4** | **P value** | **FDR p** |
| --- | --- | --- | --- | --- | --- | --- |
| Metabolism | Metabolism of terpenoids and polyketides | Biosynthesis of ansamycins | 3.73 | 3.05 | 0.033 | 0.245 |
|  | Xenobiotics biodegradation and metabolism | Drug metabolism - other enzymes | 1.21 | 1.04 | 0.018 | 0.274 |
